# Development of a Semi-Quantitative Food-Frequency Questionnaire for Korean Adults with Obesity

**DOI:** 10.3390/nu15224848

**Published:** 2023-11-20

**Authors:** Jina Chung, Seoeun Ahn, Hyojee Joung, Sangah Shin

**Affiliations:** 1 Department of Public Health Science, Graduate School of Public Health, Seoul National University, Seoul 08826, Republic of Korea; jina0126@snu.ac.kr (J.C.); hjjoung@snu.ac.kr (H.J.); 2 Institute of Health and Environment, Seoul National University, Seoul 08826, Republic of Korea; 3 Department of Food and Nutrition, Chung Ang University, Anseong 17546, Republic of Korea

**Keywords:** food-frequency questionnaire, obesity, Korea National Health and Nutrition Examination Survey

## Abstract

The increasing prevalence of obesity is a serious concern in Korea. However, there is currently no available food-frequency questionnaire (FFQ) for examining the dietary patterns of adults with obesity. This study aimed to develop a semi-quantitative FFQ tailored to Korean adults with obesity. The dish/food items for the FFQ were extracted from the 24 h recall data of 8450 Korean adults (aged 19–64 years) with obesity who participated in the 2013–2019 Korea National Health and Nutrition Examination Survey. Among the 1709 dishes consumed, 475 were selected based on their high contribution to the intake or substantial between-individual variation in 11 nutrients: energy, carbohydrates, dietary fiber, sugar, fat, saturated fat, protein, sodium, vitamin A, vitamin E, and flavonoids. These dishes were subsequently categorized into 129 items based on their recipes and primary ingredients. The final 129 items included rice; noodles and dumplings; breads, rice cakes, and cereals; soups and stews; eggs, pulses, meat, and fish; vegetables and *kimchi*; fruit; snacks; beverages; milk/dairy products; alcohol; and water. The response options for intake frequency comprised nine options, and the intake amount response included three options (50%, 100%, and 150–200% of the standard intake). After validation, this FFQ is expected to be used in epidemiological studies to investigate the dietary patterns of Korean adults with obesity.

## 1. Introduction

Around 13% of adults worldwide are obese, which is defined as having a BMI ≥ 30 kg/m^2^, and approximately 8% of annual global deaths are attributed to obesity [1]. The increasing prevalence of obesity is a serious concern in Korea. According to the World Health Organization Asia-Pacific [2] and Korean obesity standards [3,4], in which obesity is defined as a BMI ≥ 25 kg/m^2^, the prevalence of obesity among Korean adults in 2021 was 46.3% for men and 26.9% for women. Among men, the prevalence has increased by more than 10% point in the last 10 years [5].

Obesity affects nearly every organ in the human body, and is associated with a high incidence of multiple chronic conditions such as hypertension, cardiovascular disease, Alzheimer’s disease, and type 2 diabetes [6]. Its effects are not limited to individuals but also extend to society. According to a recent study that drew data from 161 countries, the economic consequences of excess weight and obesity were estimated to have accounted for 2.19% of the gross domestic product in 2019. This calculation encompassed both direct healthcare expenses and indirect costs [7].

For decades, intrinsic and extrinsic factors, such as genetics, the environment, and lifestyle, have been reported to be associated with the development of obesity. Diet is considered one of the most important factors associated with obesity [8]. Addressing the prevalence of obesity and its associated chronic conditions, as well as reducing the burden on both individuals and society, requires a focused effort to identify the dietary patterns among those affected by it.

The food-frequency questionnaire (FFQ) is a dietary survey technique employed to study an individual’s long-term dietary habits and patterns. It is distinct from open-ended dietary survey methods, such as 24 h recall and dietary records, which necessitate multiple-day surveys to estimate usual intake. It comprises dish/food items and questions related to consumption frequency. As the FFQ captures only limited intake information about the presented dish/food items, it is necessary to develop a dish/food list that accurately reflects the unique dietary characteristics of the target population.

The FFQs developed and validated for dietary surveys of Koreans include those used in the Korea National Health and Nutrition Examination Survey (KNHANES) [9,10] and the Korea Genome and Epidemiology Study (KoGES) [11,12]. However, because they were developed more than 10 years ago, these questionnaires have limitations in capturing the rapidly changing dietary patterns of Koreans. Moreover, as these studies were designed to assess the dietary intake of the general public, irrespective of the presence of diseases, they might not effectively identify dietary patterns specific to individuals with obesity. As the dietary aspects affecting disease onset and prevalence differ according to specific conditions, the use of an FFQ designed for the general population to understand the dietary behaviors of patients with specific medical conditions may not be suitable. The FFQ should be developed to adequately reflect the sources of nutrients relevant to specific diseases [13], and the dietary habits of adults with obesity may differ from those of the general population [14].

Several FFQs have been developed for research on various chronic conditions such as hypertension [15], diabetes [16,17,18,19], metabolic syndrome [20], dyslipidemia [21], and non-alcoholic fatty liver disease [22]. However, to our knowledge, no FFQ has been specifically developed for adults with obesity. Hence, creating an FFQ tailored for individuals with obesity that considers both consumption frequency and quantity can enhance the accuracy of research within this group. Consequently, the objective of this study was to develop an FFQ for Korean adults with obesity using representative dietary data gathered from individuals with obesity in Korea.

## 2. Materials and Methods

### 2.1. Data Source and Participants for Determining the FFQ Dish/Food List

To organize the dish/food list for the FFQ, we used dietary data from the KNHANES conducted year-round between 2013 and 2019. KNHANES is a nationwide cross-sectional survey conducted by the Korea Disease Control and Prevention Agency to assess the health and nutritional status of Koreans. The dietary survey of KNHANES employs a face-to-face interview method and is conducted by a trained nutritionist, who examines the type and quantity of all foods consumed by the respondents in the past 24 h [23].

Among the 44029 Korean adults who participated in the 24 h recall survey of KNHANES, we included dietary data from individuals aged 19–64 years with a BMI ≥ 25 kg/m^2^, which corresponds to obesity according to Korean obesity criteria [2,3,4]. These criteria were specifically tailored to Korean adults, aligning with the primary objective of our study to develop an FFQ for Korean adults with obesity. Subsequently, we excluded individuals with implausible energy intake (<500 kcal/d or >5000 kcal/d) as well as pregnant or lactating women.

As a result, a total of 8450 participants (4515 men and 3935 women) were selected, and they had a median age of 47 (interquartile range, 37–56) years and a median BMI of 27.1 (25.9–29.0) kg/m^2^ (Table 1).

For sugar intake data, only information from 5044 individuals (2763 men and 2281 women) who participated in the KNHANES from 2016 to 2019 was available because it was only publicly released from 2016. The KNHANES data collection process was approved by the Institutional Review Board of the Korea Disease Control and Prevention Agency, and informed consent was obtained from all participants (IRB No. 2013-07CON-03-4C, 2013-12EXP-03-5C, 2018-01-03-P-A, 2018-01-03-C-A).

### 2.2. Selection of Dish/Food List

In reviewing prior studies on foods and nutrients associated with obesity and related chronic conditions, such as high blood pressure, type 2 diabetes, cardiovascular disease, and metabolic syndrome, we identified 11 nutrient components to consider in developing the FFQ: energy, carbohydrates, dietary fiber, sugar, fat, saturated fat, protein, sodium, vitamin A, vitamin E, and flavonoids.

To construct the dish/food list for the FFQ, we initially extracted the codes of all dishes consumed by the participants. The dish codes in the raw data of the KNHANES 24 h recall survey comprise dishes/foods in their final forms, as consumed by individuals. This encompasses not only cooked dishes but also foods that individuals consume without cooking (e.g., fruit and milk). Among the entire list of dishes/foods consumed by the participants, we extracted those that were either major food sources or that exhibited substantial between-individual variation in intake for the identified 11 nutrients.

To identify the major sources of nutrients, we listed the dishes/foods in order of their contribution to nutrient intake and selected them until the cumulative contribution reached 90%. To identify the dishes/foods with high between-individual variations in intake, we conducted a stepwise regression with the individual’s daily nutrient intake as the dependent variable and the individual’s daily nutrient intake from each dish/food as the independent variable. Subsequently, the dishes/foods were extracted until the cumulative sum of the model’s explanatory power (R^2^) reached 90%.

After collecting the dishes/foods with a high intake contribution or significant between-individual variation, those consumed by less than 1% of the participants were removed. Subsequently, dishes/foods with similar main ingredients and recipes were integrated into a single group.

### 2.3. Response Options for Intake Frequency

To construct the intake frequency response options of the FFQ, we referred to the response options of previous FFQs developed for dietary surveys of Koreans, including the FFQ for KNHANES [9,10], FFQ for KoGES [11,12], and a food-oriented semi-quantitative FFQ for cancer and nutrition research [24].

### 2.4. Response Options for Portion Size

For each dish/food item, we selected the one most frequently consumed by the KNHANES participants as the representative dish. Then, we calculated the distribution (mode, median, and mean) of intake amounts per serving for these representative dishes. The reference portion sizes for these representative dishes were determined based on the calculation results and the standard portion sizes specified in the previous FFQ used in the KNHANES [11,12].

For dishes mainly consumed when eating out, the reference portion size was computed using the weight information on the dining menu from the dining-out nutritional ingredient database [25,26] and the food portion/weight database [27]. The portion size unit was expressed using the tableware typically used to serve each representative dish.

Photographs of all representative dishes were taken and included in the FFQ. A 15.5 cm long pen, an item commonly used by Koreans, was placed beside the photographed item as a reference, so that respondents could estimate the dish size.

### 2.5. Preliminary Surveys

After organizing the dish/food list and response options for intake frequency and amount, we conducted preliminary surveys to assess the comprehensibility and usability of the developed FFQ among the general population. Our participants comprised nine Korean adults, including four men and five women, ranging in age from 32 to 63 years. Participants were instructed to respond to the FFQ, and the response time was monitored. Subsequently, through surveys, we investigated the participants’ understanding of the FFQ and their difficulties in responding, and identified issues that needed to be corrected and supplemented. This survey was approved by the Institutional Review Board of Seoul National University Hospital Biomedical Research Institute (IRB No. H-2107-114-1235), and all participants provided written informed consent.

Furthermore, we collected opinions from experts, including two nutritionists and one epidemiologist, to ensure that the selection of dishes/foods for the FFQ, categorization of dish/food, and response options for intake frequency and portion size were carried out appropriately.

### 2.6. Development of a Nutrient Database for Dish/Food Items

We constructed a nutrient database for the 475 dishes/foods included in the FFQ. The recipes for each dish were composed by referencing the recipe databases published by national institutions or research institutes, including the recipe database for KNHANES [28]; the recipe database available on the MenuGen website, managed by the Rural Development Administration [29]; the recipe database for CAN-Pro of the Korean Nutrition Society [30]; and the recipe database published by the Ministry of Food and Drug Safety [25]. The nutrient contents of the foods in the recipes were referenced from the Korean Food Composition Database 10.0 [31] and the flavonoid content database for common Korean foods [32].

## 3. Results

### 3.1. Dish/Food List of the FFQ

Among the 1709 dishes/foods consumed by the 8450 participants, 827 were extracted, after excluding those with low intake contribution rates and small between-individual variations in nutrient intake. Of these 827 dishes/foods, 552 were eliminated because they were consumed by less than 1% of the participants. The remaining 275 dishes/foods were subsequently categorized based on their recipes and main ingredients. For instance, dishes such as ham sandwiches, ham and cheese sandwiches, chicken sandwiches, hamburgers, and *bulgogi* burgers were grouped into the item ‘sandwich and hamburger’.

The dish/food list was further refined, and 200 foods/dishes were reintroduced. This reintroduction comprised dishes/foods that had been removed because of consumption by less than 1% of the participants, yet their intake contribution rate or between-individual variation was within 50% for any of the nutrients. Moreover, we reintroduced dishes/foods that were often linked to specific item names. For instance, when the term ‘grilled beef’ naturally evoked thoughts of not only Korean-style ‘grilled beef’ but also western-style ‘steak’, we included these items in the detailed menu, leading to a modification in the item name to ‘grilled beef, steak’. Additionally, we included ‘water’ as the last dish/food item.

Finally, 475 dishes/foods were selected; their cumulative intake contribution by nutrient ranged from 74.9–85.2% (average: 80.6%), and their cumulative explanatory power of between-individual variation ranged from 43.6 to 86.9% (average: 74.2%) (Table 2).

These 475 dishes/foods were categorized into 129 items based on their recipes and main ingredients. Finally, these 129 items were listed in the FFQ in the same order as presented in Table 3. Detailed information on individual dish/food items can be found in Table S1.

### 3.2. Question and Response Options for Intake Frequency

The reference period for the survey was set at 1 year. For each item, respondents were asked, “How often did you eat the dish on average in the past year?” The response options were categorized into nine levels: rarely, once a month, 2–3 times a month, 1–2 times a week, 3–4 times a week, 5–6 times a week, once a day, twice a day, and 3 times a day.

For the coffee categories, which were likely to be consumed more than three times a day, we added short-answer questions as follows: “If you drank coffee more than 3 times a day, how many times per day did you consume it on average?”.

Regarding the fruit category, we asked whether the fruit was consumed only in season or throughout the year, before asking about the intake frequency. If the respondents answered that they ate the fruit only in season, they were asked to answer the subsequent intake frequency question using the average frequency of consumption during the season. However, if the respondents answered that they consumed it regardless of the season, they were asked to indicate the average frequency of consumption throughout the year.

### 3.3. Question and Response Options for Portion Size

**Figure 1:**
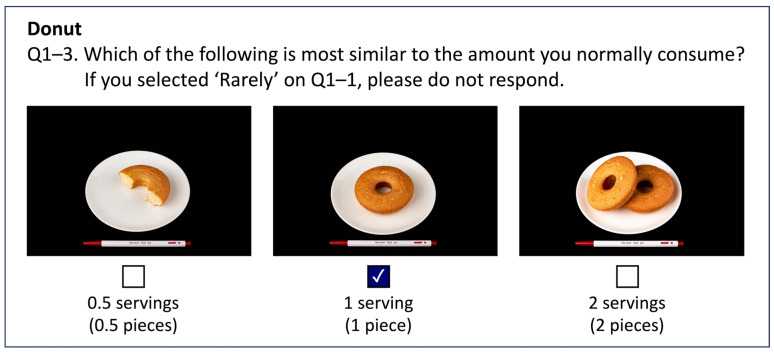
Example of the questionnaire on portion size.

To investigate the intake amounts for each dish/food item, we posed the question, “Which of the following is most similar to the amount you normally consume?” Three response options were provided: small (standard portion size × 0.5), medium (standard portion size × 1.0), and large (standard portion size × 1.5). Each response option was accompanied by images of representative dishes (Figure 1).

The standard portion size was determined based on the intake amount per serving of the corresponding dish/food among KNHANES participants. For instance, the modal and median volume of milk consumed per serving was 200 mL; consequently, the standard portion size for milk was established at 200 mL.

An ‘extra-large (standard portion size × 2.0)’ portion size option was added for dishes where rice served as the main ingredient, such as rice (excluding multigrain rice), multigrain rice (including rice with beans), and *gimbap*.

Regarding alcoholic beverages, additional clarification was included after the portion size question: “If your alcoholic beverage intake per serving surpasses the largest size provided, please specify your average intake amount”.

### 3.4. Preliminary Survey and Revision of the FFQ

The average time taken by the nine participants to complete the FFQ was 31.4 min (range, 18–48 min). When asked about the appropriateness of the number of dish/food items, seven out of nine participants responded that it was appropriate, while two felt that the number of items was small. Regarding the response option for intake frequency, all participants agreed that it was appropriately structured. As for the response option for portion size, eight participants stated that response options or photographs of dishes were appropriate, and one participant noted that the number of response categories and photographs was insufficient.

In addition, we identified certain aspects that the participants found challenging to understand and subsequently refined them through expert consultations. For instance, in the coffee category, the initial question asked about the intake frequency and amount of ‘instant coffee’ and ‘brewed coffee’ without further explanation. However, participants encountered difficulties in distinguishing between the two. Reflecting these opinions, we added an example after the food name to provide clarification: instant coffee (e.g., mixed coffee, canned coffee) and brewed coffee (e.g., black coffee, café latte).

Furthermore, we included subsequent questions regarding the types of coffee typically consumed. For instant coffee (e.g., mixed coffee, canned coffee), the response options included (1) black, (2) mix, and (3) both. For brewed coffee (e.g., black coffee, café latte), the response options included (1) black (without syrup), (2) café latte (without syrup), (3) black with syrup, and (4) café latte with syrup.

### 3.5. Nutrient Database for the FFQ

We developed the nutrient database for the FFQ by formulating recipes for the 475 dishes/foods included in the questionnaire. These recipes were then linked with a nutrient database containing information on the nutrient content of each food item. In cases where a dish/food item comprised multiple dishes, we calculated the nutrient content by multiplying each dish’s nutrient values by a weight determined according to the frequency of intake for each food. These values were then summed for all dishes. The intake frequency weights for each dish were determined based on data from Korean adults with obesity who participated in the 2013–2019 KNHANES.

For example, the yogurt item included both yogurt and liquid-type yogurt, each having an energy content of 89.0 kcal and 68.3 kcal for the standard portion, respectively. The KNHANES participants reported consuming yogurt 921 times and liquid-type yogurt 308 times. As a result, we calculated intake frequency weights of 0.75 and 0.25, respectively. Consequently, the energy content of the yogurt item was determined as 89.0 kcal multiplied by 0.75 plus 68.3 kcal multiplied by 0.25, resulting in 83.8 kcal.

## 4. Discussion

The prevalence of obesity among Korean adults is on the rise. Diet plays a crucial role in the pathogenesis of obesity [33]. Therefore, there is a need to develop tools for assessing long-term dietary patterns and identifying the specific dietary factors associated with obesity. In particular, the semi-quantitative FFQ is a widely used method in epidemiological studies to establish associations between diet and chronic diseases because it can approximate respondents’ food and nutrient intakes in a relatively simple way.

In this study, we developed a semi-quantitative FFQ for Korean adults with obesity using recent nationally representative epidemiological data. The FFQ comprised 475 dishes/foods out of the 1709 dishes/foods consumed by 8450 Korean adults with obesity who participated in the 2013–2019 KNHANES. The energy and nutrient intakes derived from these dishes/foods accounted for 80.6% of the total intake and explained 74.2% of the between-individual variation.

These 475 dish/food items were combined into 129 items by grouping those with similar recipes and main ingredients. Specifically, in this study, ‘water’ was included as one of the FFQ dish/food items, considering the decreasing proportion of Koreans meeting their recommended water intake [34]. The number of items in this FFQ exceeded that of other FFQs used in large-scale epidemiological studies in Korea, such as the FFQs for the KNHANES (112 items) and the KoGES (112 items). However, it remained within the typical range of FFQ item counts, ranging from 50 to 200 items [13].

In Korea, the consumption of ultra-processed foods is increasing [35]. This trend is concerning because an increased intake of ultra-processed foods leads to higher intakes of energy, sugar, fat, and saturated fat while reducing essential dietary components such as dietary fiber, vitamins, and minerals [36,37]. Additionally, previous studies conducted on Koreans found that a higher intake of ultra-processed foods is associated with an increased prevalence and incidence of obesity [38,39,40,41]. Consequently, in the process of selecting items for the FFQ dish/food list, we carefully considered nutritional components, such as dietary fiber, sugar, fat, and saturated fat, which are known to be associated with ultra-processed foods. As a result, our FFQ includes approximately 20 ultra-processed food items, including soft drinks, cookies, and sausages.

This FFQ was designed to enable respondents to indicate their intake frequency over the past year, using one of nine options for each dish/food item. This structure is consistent with the FFQs of the KNHANES [9,10] and KoGES [11,12]. However, several exceptions were made considering the dietary habits of Koreans. In the coffee category, the consumption of which is increasing among Korean adults [42,43], we designed the questionnaire to instruct respondents to specify their actual intake frequency in a short-answer format if they consumed coffee more than three times a day. Additionally, we included extra questions to address the intake frequency in the fruit category. We aimed to facilitate the respondents’ recall and response regarding fruit intake by first inquiring whether they consumed the fruit only during the season or year-round, regardless of the season. Subsequently, we asked participants about their intake frequency during the specified response period.

This FFQ was developed as a semi-quantitative tool, offering three-to-four response options for the portion size to enhance the accuracy of estimating the respondent’s food and nutrient intake. The semi-quantitative FFQ is known for its ability to more accurately assess food and nutrient intake, partially reflecting the variations in amounts consumed between individuals compared to a general FFQ without portion size questions [44]. Expanding on this, we implemented further efforts to enhance the accuracy of the intake estimation. First, considering that individuals with obesity might have different average portion sizes from individuals without obesity, we determined standard portion sizes by considering the mode, median, and mean amounts consumed per occasion for each food item, using 24 h recall data from Korean adults with obesity. Second, the Korean diet relies heavily on rice, a carbohydrate-rich staple food, and excessive carbohydrate intake is a major contributor to obesity [45]. Therefore, an ‘extra-large’ portion size option was added for dishes including rice (excluding multigrain rice), multigrain rice (including rice with beans), and *gimbap*. Third, within the soups and stews category, distinct standard amounts were assigned to individual items. For instance, for soups and stews typically consumed at home, the standard amount was determined based on the size of household soup bowls. Conversely, for soups and stews that are primarily consumed when dining out, the standard portion size was based on the bowl size commonly used in restaurants. Fourth, for alcoholic beverages, where individual variations in intake amount were significant, respondents were instructed to specify their average intake amount if it exceeded the provided ‘extra-large (standard portion size × 2.0)’ portion size. Finally, photographs of all food items and their respective portion sizes were included. This comprehensive and diverse approach is expected to result in more accurate measurements of respondents’ food and nutrient intake.

Through a preliminary survey conducted with nine non-experts, we ascertained that the general population could comprehend the structure and response methodology of this FFQ. The questionnaire was augmented with expert advice to address certain challenging aspects. For instance, specific examples were incorporated based on feedback from participants who encountered difficulties in distinguishing between different coffee items. Additionally, a supplementary question was introduced regarding the type of coffee consumed, to achieve a more precise estimation of the respondents’ sugar and milk intake.

The FFQ developed in this study is the first specifically designed for Korean adults with obesity. The FFQ development method used in this study can be applied to other studies developing FFQs for other chronic diseases. The FFQ developed in this study possesses notable strengths, as it was developed using representative national epidemiological data. Furthermore, it reflects the contemporary dietary patterns of Korean adults because it is based on recent dietary survey data. Additionally, because the KNHANES is conducted year-round, it enables a comprehensive assessment of dietary patterns across all seasons.

A limitation of our study is that the dietary data from the KNHANES, which were used to select the FFQ dish/food list, were collected through a single day’s 24 h dietary recall. Consequently, the dietary survey results may not fully reflect the usual intake patterns of each participant. However, the substantial number of participants extracted to represent the entire Korean population compensated for this limitation.

Our research team plans to validate the accuracy and reproducibility of the FFQ developed in this study. After a validity and reliability verification, this FFQ could potentially be used to examine dietary patterns among individuals with obesity in epidemiological studies. In addition, by identifying dietary factors related to obesity, we believe that it will be possible to lay the foundation for the establishment of nutritional policies and projects for the prevention of chronic diseases. Ultimately, we anticipate that this will help reduce the socioeconomic costs associated with the management and prevention of obesity and other chronic diseases.

## Figures and Tables

**Table 1:** Distribution of age and BMI in participants whose dietary data were used for dish/food list selection.

	Total (*n* = 8450)	Men (*n* = 4515)	Women (*n* = 3935)
Median age (years)	47 (37–56) ^1^	44 (35–55)	50 (40–58)
Age distribution, *n* (%)			
19–29 years	960 (11.4)	630 (7.5)	330 (3.9)
30–49 years	3783 (44.8)	2202 (26.1)	1581 (18.7)
50–64 years	3707 (43.9)	1683 (19.9)	2024 (24.0)
Median BMI (kg/m^2^)	27.1 (25.9–29.0)	27.0 (25.9–28.8)	27.1 (26.0–29.1)
BMI distribution, *n* (%)			
25 to <30 kg/m^2^	7068 (83.6)	3856 (45.6)	3212 (38.1)
30 to <35 kg/m^2^	1169 (13.8)	562 (6.7)	607 (7.2)
≥35 kg/m^2^	213 (2.5)	97 (1.2)	116 (1.4)

^1^ Values are medians ± interquartile ranges for continuous variables and *n* (%) for categorical variables.

**Table 2:** The cumulative intake contribution rate and cumulative R^2^ for between-individual variation from the selected 475 dishes/foods for each nutrient.

	Cumulative Contribution Rate (%)	Cumulative R^2^ of between-Individual Variation
Energy	84.4	81.2
Carbohydrate	85.2	79.3
Sugar	83.2	86.9
Dietary fiber	82.6	83.0
Protein	79.2	73.3
Fat	80.3	81.2
Saturated fat	82.5	83.9
Sodium	78.3	77.0
Vitamin A	74.9	52.2
Vitamin E	78.9	75.2
Flavonoids	77.2	43.6
Average	80.6	74.2

**Table 3:** The 12 categories of FFQ’s dish/food list and number of dish/food items in each category.

Dish/Food Category (*n* = 12)	Numbers of Dish/Food Items (*n* = 129)
Rice	7
Noodle and dumplings	*6*
Bread, rice cakes, and cereal	10
Soup and stews	12
Eggs, pulses, meats, and fishes	28
Vegetable and *kimchi*	31
Fruits	14
Snack	4
Milk and dairy products	4
Beverage	9
Alcohol	3
Water	1

## Data Availability

Publicly available datasets were analyzed in this study. This data can be found here: https://knhanes.kdca.go.kr/knhanes/sub03/sub03_02_05.do.

## Supplementary Materials

The following supporting information can be downloaded at: https://www.mdpi.com/article/10.3390/nu15224848/s1, Table S1: The 129 dish/food items of the FFQ.
