# Supplementary material for: Development of a Semi-Quantitative Food-Frequency Questionnaire for Korean Adults with Obesity
Source: Nutrients. 2023 Nov 20;15(22):4848. doi: 10.3390/nu15224848 (PMC10675269; doi:10.3390/nu15224848)
Supplement: Supplementary file 1 [file nutrients-15-04848-s001.zip › nutrients-2674327-supplementary.pdf]

nutrients MDPI

Title: Development of a Semi-Quantitative Food-Frequency Questionnaire for Korean Adults with Obesity

Table S1. The 129 dish/food items of the FFQ

Category	Dish/Food Items
Rice (n = 7)	- Rice (excluding multigrain rice)<br>- Multigrain rice (including rice with beans)<br>- Fried rice, *bibimbap* (cooked rice with assorted mixtures)<br>- Curry rice, *jajangbap* (rice with black soybean sauce)<br>- *Gimbap* (rice rolled in laver), *jumeokbap* (rice balls)<br>- Sushi<br>- Porridge
Noodle and dumplings (n = 6)	- *Ramyeon* (instant noodles), *cup ramyeon* (instant cup noodles)<br>- Noodles (including noodles mixed with spices), *kalguksu* (noodle soup), udon<br>- *Jajangmyeon* (black bean sauce noodles), *jjamppong* (spicy seafood noodle soup)<br>- *Naengmyeon* (cold buckwheat noodles)<br>- *Tteok-guk* (sliced rice cake soup), *Tteongmandu-guk* (sliced rice cake and dumpling soup)<br>- *Mandu* (steamed or fried dumpling)
Bread, rice cakes, and cereal (n = 10)	- Loaf bread, bagel, diner roll<br>- Red bean bun (sweet red bean bun, steamed sweet red bean bun), cream buns, jam bread<br>- Hot dog, sausage bread, croquette<br>- Donuts, twisted bread sticks, pastries, croissants and pies<br>- Cake, choco pie, sponge cake (castella)<br>- Pizza<br>- Sandwich, hamburger<br>- *Injeolmi* (cubed rice cake with soybean powder), *jeolpyeon* (plain cubed rice cake), *baeksulgi* (plain steamed rice cake), *sirutteok* (steamed rice cake with red bean)<br>- *Tteok-bokki* (stir-fried rice cake)<br>- Cereal
Soup and stews (n = 12)	- *Sundae-guk* (Korean sausage soup), *gamja-tang* (pork backbone stew), *haejang-guk* (hangover soup), *chueo-tang* (loach soup)<br>- *Sagol-guk* (beef bone soup), *galbi-tang* (short rib soup), *gom-tang* (beef bone soup), *seolleong-tang* (ox bone soup)<br>- *Yukgaejang* (spicy beef soup)<br>- *Miyeok-guk* (seaweed soup)<br>- *Mu-guk* (radish soup), *sojogi-mu-guk* (beef and radish soup)<br>- *Bugeo-guk* (dried pollack soup)<br>- *Doenjang-guk* (soybean paste soup)<br>- *Budae-jjigae* (spicy sausage stew)<br>- *Dongtae-jjigae* (frozen pollack stew), *haemul-maewoon-tang* (spicy seafood stew)<br>- *Doenjang-jjigae* (soybean paste stew), *cheonggukjang-jjigae* (rich soybean paste stew)<br>- *Dubu-jjigae* (bean curd stew), *sundubu-jjigae* (soft bean curd stew)<br>- *Kimchi* stew, stir-fried *kimchi*, *kimchi* soup
Eggs, pulses, meats, and fishes (n = 28)	- Grilled pork belly<br>- Grilled pork excluding pork belly (ribs, skirt steak, etc.)<br>- *Jeyuk bokkeum* (Spicy stir-fried pork), pork *bulgogi*, pork *galbi-jjim* (braised pork rib), *jokbal* (braised pigs' feet)<br>- *Tangsuyuk* (sweet and sour pork), pork cutlet<br>- Grilled beef, steak<br>- Beef *bulgogi*, beef *galbi-jjim* (braised beef rib), beef *bokkeum* (stir-fried beef)<br>- *Tteokgalbi* (grilled short rib patties), *wanja-jeon* (pan-fried meatball)

	- Ham, sausage<br>- *Sundae* (Korean sausage)<br>- *Samgye-tang* (ginseng chicken soup)<br>- *Dakbokkeum-tang* (spicy braised chicken), *dakgalbi* (spicy stir-fried chicken), grilled chicken<br>- Chicken (fried chicken)<br>- Grilled duck<br>- Fried egg, egg *mal-i* (rolled omelet)<br>- Boiled egg, steamed egg<br>- Bean curd, Bean curd *jorim* (braised bean curd), Bean curd *buchim* (pan-fried bean curd)<br>- *Kongjorim* (braised soybean in soy sauce)<br>- Mackerel, saury (grilled, braised)<br>- Hairtail, croaker (grilled, braised)<br>- Pollack (*dongtae jeon* (pan-fried battered pollack fillet), *myeongtae jjim* (braised pollack), *myeongtae gui* (grilled pollack), etc.)<br>- Grilled eel<br>- *Hoe* (sliced raw fish)<br>- Anchovies, Anchovies *bokkeum* (stir-fried dried anchovies)<br>- Squid (raw, boiled, spicy stir-fried), dried shredded squid (stir-fried, seasoned), dried squid<br>- Shrimp (fried shrimp, grilled shrimp, etc.)<br>- *Gejang* (crab seasoned with soy sauce or spicy sauce)<br>- Shrimp *jeot* (salted shrimp), squid *jeot* (salted squid), pollack roe *jeot* (salted pollack roe), octopus *jeot* (salted octopus)<br>- *Eomuk bokkeum* (stir-fried fish cake), *eomuk guk* (fish cake soup)
Vegetable and *kimchi* (n = 31)	- Korean cabbage *kimchi*<br>- Other *kimchi* (except for Korean cabbage *kimchi*)<br>- Bean sprout *muchim* (bean sprout salad), bean sprout *guk* (bean sprout soup), *sukju namul* (mung bean sprout salad)<br>- Spinach *namul* (spinach salad)<br>- Green vegetable salad<br>- Other vegetable salad<br>- Green onion *muchim* (green onion salad), chive *muchim* (chive salad)<br>- Cucumber (fresh salad, raw)<br>- Vegetable salad<br>- Boiled cabbage, boiled broccoli<br>- Leaf wraps (lettuce, sesame, cabbage, pumpkin leaf)<br>- Green pepper<br>- Garlic<br>- Onion<br>- Carrot<br>- *Ssamjang* (dipping sauce for wraps), *gochujang* (red chili paste), *doenjang* (soybean paste), *cho-go-chujang* (vinegar and red chili paste)<br>- Pickled radish, radish fresh salad, dried radish<br>- Pickled vegetables (sesame leaf, radish, chili pepper, onion, garlic), pickled cucumber<br>- Lotus roots-*jorim* (lotus roots boiled with soy sauce), burdock-*jorim* (burdock boiled with soy sauce)<br>- Mushroom *bokkeum* (stir-fried mushroom)<br>- *Jeon* (chive pancake, *kimchi* pancake pumpkin pancake)<br>- *Japchae*<br>- *Miyeok julgi bokkeum* (stir-fried seaweed stems), *miyeok cho muchim* (sweet and sour seaweed salad), *parae muchim* (green laver salad)<br>- *Kim gui* (grilled laver), *saeng kim* (raw laver), *kim muchim* (laver salad)

	- Steamed potatoes, grilled potatoes<br>- Potato *jorim* (braised potatoes in soy sauce), potato *bokkeum* (stir-fried potatoes)<br>- French fries<br>- Steamed sweet potatoes, grilled sweet potatoes<br>- Steamed corn, grilled corn<br>- Chestnut<br>- Peanut, almond, walnut
Fruits<br>(n = 14)	- Strawberry<br>- Tomato, cherry tomato<br>- Melon<br>- Watermelon<br>- Peach<br>- Grape<br>- Apple<br>- Pear<br>- Persimmon, dried persimmon<br>- Banana<br>- Mandarin, orange<br>- Kiwi<br>- Plum<br>- Pineapple
Snacks<br>(n = 4)	- Snack<br>- Cookie, cracker<br>- Candy<br>- Chocolate
Beverage<br>(n = 9)	- Instant coffee (e.g., mixed coffee, canned coffee)<br>- Brewed coffee (e.g., black coffee, café latte)<br>- Green tea, black tea<br>- Other teas (barley tea, corn tea, corn silk tea, mugwort tea, Solomon's seal, herbal tea, etc.)<br>- Teas containing sugar (Job's tears tea, *yuja tea* (Korean citron tea), ginger tea, ginseng tea, etc.)<br>- Soft drinks (cola, soda, fruit juice soda)<br>- Fruit juice, vegetable juice<br>- *Misutgaru* (mixed grain powder beverage), *sikhye* (sweet rice punch)<br>- Other beverages (vitamin drinks, sports drinks)
Milk and dairy products<br>(n = 4)	- Milk (normal, low fat)<br>- Soybean milk<br>- Yogurt (curd type, liquid type)<br>- Ice cream, ices
Alcohols<br>(n = 3)	- *Soju*<br>- Beer<br>- *Makgeolli* (unrefined rice wine)
Water<br>(n = 1)	- Water
